# The changes of Th17 cells and the related cytokines in the progression of human colorectal cancers

**DOI:** 10.1186/1471-2407-12-418

**Published:** 2012-09-21

**Authors:** Jiansheng Wang, Kaiyu Xu, Jing Wu, Chenghua Luo, Yuchen Li, Xuebin Wu, Hong Gao, Guosheng Feng, Bao-Zhu Yuan

**Affiliations:** 1 Department of Oncology, Beijing Shijitan Hospital, Capital Medical University, Beijing, China, 100038; 2 Department of Cell Biology, Institute for Biological Products Control, National Institutes for Food and Drug Control, Beijing, China, 100050; 3 Beijing Chao-Yang Hospital, Capital Medical University, Beijing, China, 100020; 4 Present address: Clinical Laboratory, Hebei General Hospital, Shijiazhuang, Hebei, China, 050051

**Keywords:** Colorectal adenoma, Colorectal cancer, Th17 cells, Treg cells, Cytokines

## Abstract

**Background:**

The role of Th17 cells in colorectal tumorigenesis and development still remains unclear, despite the fact that it has been established in the pathogenesis of autoimmune diseases.

**Methods:**

We first analyzed Th17 cells and Treg cells using flow cytometry in the circulation of colorectal adenoma (CRA) and colorectal carcinoma (CRC) patients and healthy controls, and the frequency of Th17 cells in peripheral blood mononuclear cells (PBMCs) stimulated by anti-CD3 plus anti-CD28 and treated by IL-1β, IL-6, and TGF-β in different concentrations. We then detected cytokines IL-1β, IL-6, IL-17A, IL-21, IL-23 or TGF-β by ELISA in sera and supernatants from both normal and tumor tissues cultured *ex vivo*.

**Results:**

It was found that the percentage of Th17 and Treg cells increased in the circulation of both CRA and CRC patients; the increase of Th17 cells in the circulation occurred in early stages, whereas the increase of Treg cells in the circulation and the increase of Th17 cells in tumor tissues occurred in advanced stages. The subsequent cytokine profiling showed that, along CRC progression, IL-1β, IL-17A and IL-23 underwent a similar change, while IL-6 in CRC exhibited an opposite change, with Th17 cells. In addition, high levels of TGF-β and IL-17A were detected in tumor tissues rather than in normal mucosa. The *in vitro* experiment further demonstrated that IL-1β, IL-6 or TGF-β modulated Th17 cell expansion in PBMC.

**Conclusions:**

Our study reveals a unique change of Th17 cells, which is regulated possibly by IL-1β, IL-6 and TGF-β in the progression of CRC.

## Background

Colorectal cancer (CRC) is one of the leading causes of cancer death in the world. Although the modality of CRC has recently showed a slow reduction due to improvement in early detection and/or treatment in the United States, it still keeps rising in China, especially in the big metropolitan areas [1,2], thus still imposing a severe threat to human health.

According to the adenoma-carcinoma sequence hypothesis [3], CRC develops through a multistep process from initially low-grade dysplastic adenoma to high-grade dysplastic adenoma and ultimately to carcinoma. Progression through this sequence is accompanied by accumulation of tumorigenesis-related genetic mutations and molecular changes [3,4]. The increasing evidence shows that the balance in different CD4-positive T lymphocyte subpopulations and cytokine networks are also deregulated [4-6]. The changes of cytokine profiles in the serum may be early events along the colorectal adenoma-carcinoma sequence. Thus, they may serve as important biological indices for the prognosis of CRC [6-8]. In addition, subtyping of immune cells within tumor tissues may be more effective in evaluating disease status than genetic changes [9-11].

Among different T lymphocyte subpopulations, CD4^+^CD25^+^ T cells (Tregs) are vital in maintaining immune tolerance and homeostasis. But they may promote tumor progression by inhibiting anti-cancer immune responses [12,13]. Recent evidence suggests that expression of IL-7 receptor-chain (also known as CD127) on cell surface is inversely correlated with the suppressive function of CD4^+^CD25^hi^ Tregs and the expression of Foxp3, which is an Treg-expressing transcription factor, so the Tregs can also be characterized by CD4^+^CD25^+^CD127^lo/-^ phenotype [14,15].

Recently, the Th17 cell, which is another CD4-positive T lymphocyte subpopulation with its designation following the feature of secreting IL-17, has been extensively described for its involvement in several autoimmune diseases and chronic inflammatory syndromes [16,17]. However, the role of Th17 cells in tumor immunity was less studied as compared with Treg cells.

Among all cytokines, IL-1β, IL-6, IL-17, IL-21, IL-23 and TGF-β have been implicated in several autoimmune diseases and inflammatory disorders. They are also known to be able to modulate differentiation and development of Th17 cells and Treg cells [17,18]. In mice, low doses of TGF-β, together with IL-6 or IL-21 can initiate differentiation of naïve CD4^+^ T cells into the Th17 lineage, while high doses of TGF-β can drive formation of the Treg lineage from naïve CD4^+^ T cell [19,20]. IL-23 is thought to be required in the maintenance of Th17 cell phenotype [17,19,21,22]. In humans, in addition to IL-6, IL-21, TGF-β and IL-23,IL-1β was also found important in regulating Th17 cell differentiation [22-26].

Despite a great knowledge about differentiation and function of Th17 cells in both mice and humans has been accumulated, generation and regulation of Th17 cells in human cancer, especially in colorectal cancer, still remain unclear. In this study, we investigated the changes of Th17 and Treg cells, and the related cytokines in the peripheral blood of patients with colorectal adenoma (CRA) or CRC. We also detected the frequency of Th17 cells and concentrations of the related cytokines in both CRC tissues and the surrounding normal tissues. In addition, to determine which cytokines play more dominant roles in the prevalence of Th17 cells, we subsequently assessed in an *in vitro* experiment the effect of the related cytokines on the expansion of Th17 cells from peripheral blood mononuclear cells (PBMCs).

## Methods

### Patients

Sixty six patients of 35 sporadic colorectal cancers and 31 colorectal adenomas, admitted in Beijing Shijitan Hospital from June 2010 to July 2011, were enrolled in this study. Both CRA and CRC patients were divided into two groups based on disease progression. The findings of tubular I and tubular II correspond to early stage and late stage, respectively, for CRA patients; the stage I/II and stage III/IV were considered as early and late stage, respectively for CRC patients. None of them received radiotherapy, chemotherapy, or immunotherapy before sample collection. The subjects with cancer history, autoimmune diseases, infectious diseases, inflammatory bowel diseases and familial polyposis were excluded in this study. Patient profiles are summarized in Table 1. Twenty four healthy donors without history of tumor or other serious illnesses, in which 12 are female and 12 are male with mean age at 63 ranging from 42 to 83, were included as controls. The sample collection was performed with a written informed consent for each subject and the study was conducted with the approval of the Shijitan Hospital’s ethics committee and in compliance with the Helsinki Declaration.

**Table 1:** Clinical features of patients with colorectal adenoma and carcinoma

	**Colorectal adenocarcinoma**	**Colorectal adenoma**
Sex		
Male	15	21
Female	20	10
Age, y		
Median	64	67
Range	43-86	45-83
Location		
Right side of colon	5	6
Transverse colon	4	3
Left side of colon	3	5
Sigmoid colon	8	10
Rectum	15	7
Adenocarcinoma differentiation		
Well	8	
Moderate	20	
Poor	7	
AJCC cancer stage		
I	4	
II	10	
III	17	
IV	4	
Adenoma histological grade		
TubularI		19
TubularII		12

### Reagents

Collagenase type IV, hyaluronidase, deoxyribonuclease type I, Ficoll-Hypaque and Percoll were from Sigma-Aldrich. Recombinant IL-1β, IL-6 and TGF-β1 were from PeproTech. Anti-human CD4, CD25 and isotype controls were from BD Bioscience. Phorbol 12-myristate 13-acetate (PMA), ionomycin, Brefeldin A, FIX & PERM Kit Reagent, IL-1β, IL-6, IL-17A, IL-21, IL-23, TGF-β ELISA kit, and Anti-human CD3, CD28, CD8, CD127, IL-17A and isotype controls were from eBioscience.

### Tissue culture

Tissue culture was performed as previously described [27]. Briefly, tumor tissue or normal mucosa was washed, weighed, and then placed in a small tissue culture dish containing RPMI-1640 medium supplemented with 10% FBS and antibiotics. After incubation at 37°C with 5% CO2 for 24 h and then centrifugation, the supernatant of each sample was used for cytokine measurements.

### Cell isolations

Normal infiltrating lymphocytes (NILs) and tumor infiltrating lymphocytes (TILs) were isolated from freshly resected surgical specimens by a method reported previously with some modifications [28]. In brief, the epithelial layer was removed by stirring in 1 mM EDTA and 1 mM DTT for 1 h at 37°C. The tissue was minced, then treated with a digestion solution containing 0.5 mg/ml collagenase type IV, 1 mg/ml hyaluronidase and 0.1 mg/ml deoxyribonuclease type I for 2 h with stirring at 37°C. After digestion, the cells were washed and centrifuged over a discontinuous Percoll gradient (75% and 40%). PBMCs were isolated using Ficoll-Hypaque gradient. The cells at the interface were harvested, washed and re-suspended in RPMI-1640 complete medium. The cell viability was determined by trypan blue exclusion.

### Stimulation of PBMCs

The freshly isolated PBMCs (1 × 10^6^ cells/well) from health donors were stimulated in 4 μg/ml anti-CD3 monoclonal antibody-coated 96-well plate and 2 μg/ml anti-CD28 antibody, and incubated for 84 h with RPMI-1640 complete medium containing different cytokines (IL-1β, 10 and 25 ng/ml; IL-6, 25 and 50 ng/ml; TGF-β1, 5 and 0.5 ng/ml ) alone or in combination. For intracellular cytokine staining, the purified PBMCs, NILs and TILs were stimulated for 5 h in RPMI complete medium with 50 ng/ml PMA and 1 μg/ml ionomycin in the presence of 10 μg/ml Brefeldin A.

### Cytokine measurements

Concentrations of cytokines IL-1β, IL-6, IL-17A, IL-21, IL-23 or TGF-β in sera, supernatants of tissue, and cell cultures of the stimulated PBMCs were measured using a specific ELISA kit. Results of the cytokine concentrations in the supernatant of tissue cultures are expressed as pg/mg of tissue weight/ml of culture medium.

### Flow cytometry assays

Surface protein staining of PBMCs, NILs and TILs were performed at room temperature for 20 min using the following antibodies: the PE-Cy5-conjugated anti-CD3 and FITC-conjugated anti-CD8 were used for staining Th17 cells; the FITC-conjugated anti-CD4 and PE-Cy^TM^7-conjugated anti-CD25 and PE-conjugated anti-CD127 were used for Treg Cells. Before staining with the PE-conjugated anti-IL-17A, cells were washed, fixed and permeated using FIX & PERM Kit Reagent following the manufacturer’s instructions. The isotype control of each dye-conjugated antibody was used to correct compensation and confirm antibody specificity. The stained cells were analyzed by a Calibur flow cytometer equipped with CellQuest software.

### Statistical analysis

Data were analyzed by SPSS statistical software (version 16, SPSS Inc., Chicago, IL) using Student's t test or one-way ANOVA and the Mann–Whitney U test or the Kruskal-Wallis test. A level of *P <* 0.05 was considered statistically significant.

## Results

### Percentage of the circulating Th17 cells in CRA and CRC patients

**Figure 1:**
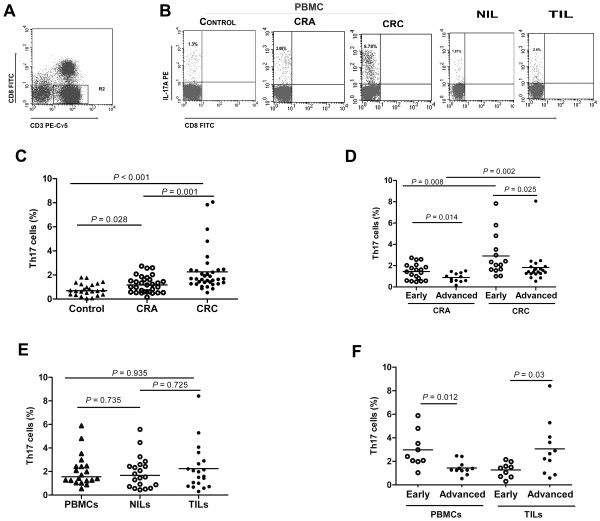
**The population of Th17 cells in PBMCs , NILs and TILs.A**. The CD3^+^CD8^-^ T (CD4^+^ T) cells were gated after lymphocytes gating. **B**. Proportions of Th17 cells to CD4^+^ T subsets from the representative PBMC, NIL and TIL were indicated. Percentages of Th17 cells are shown in CRA (n = 31), CRC (n = 35), and healthy donors (n = 24) (**C**); in patients at early (CRA, n = 19; CRC n = 14) and advanced (CRA, n = 12; CRC n = 21) stages (**D**); in PBMCs (n = 20), NILs (n = 20) and TILs (n = 20) from the same CRC patients (**E**), and in PBMCs and TILs from the same patients at early (n = 9) and advanced (n = 11) stages (**F**). Each datum point represents an individual sample. The horizontal line represents median values for each group.

To investigate the changes of Th17 cells in the progression of CRC, we first measured Th17 cells in the PBMCs of CRA and CRC patients and healthy controls. The analysis for the percentage of Th17 cell in total CD4^+^T cells in patients and controls were shown in Figure 1. The percentage of Th17 cells was significantly increased in both CRA (median, 1.2%; 25^th^ to 75^th^ percentiles, 0.6% to 1.6%; *P =* 0.028) and CRC patients (1.7%; 1.3% to 2.4%; *P <* 0.001) as compared with healthy controls (0.7%; 0.4% to 1.2%). In addition, the percentage of Th17 cells in CRC patients was significantly higher than that in CRA patients (*P =* 0.001). Following disease progression, we observed that the percentage of Th17 cells was decreased in advanced stages of both CRAs and CRCs in comparison with early stage of each disease (0.8%; 0.5% to 1.3% vs 1.4%; 0.6% to 2.0%, *P =* 0.014; 1.4%; 1.3% to 1.9% vs 2.2%; 1.6% to 3.8%, *P =* 0.025). Although both in advanced stages, CRC patients showed a higher percentage of Th17 cells than CRA patients (*P =* 0.002).

### Distribution of Th17 cells in NILs and TILs

Subsequently, we found that percentage of Th17 cells in TILs (1.8%; 0.8% to 2.8%) was increased as compared with NILs (1.7%; 0.8% to 2.6%) or PBMCs (1.6%; 1.2% to 2.5%), but did not show any statistical significance. However, the percentage of Th17 cells in the TILs of patients in advanced stages was significantly higher than that in early stages (2.7%; 1.0% to 4.1% vs 1.4%; 0.7% to 1.8%, *P =* 0.03) (Figure 1).

### Percentage of the circulating Tregs in CRA and CRC patients

**Figure 2:**
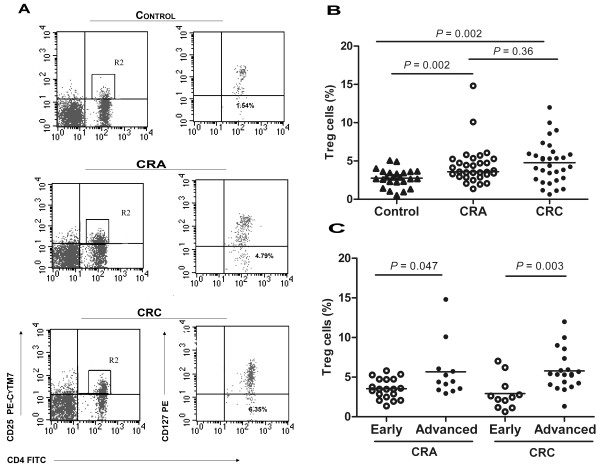
**Population of the Treg cells in PBMCs.A**. CD4^+^CD25^+^ T cells were gated after lymphocytes gating and the calculated numbers of CD4^+^CD25^+^CD127^-^ T cells in CD4^+^ T cells were indicated in the representative control, CRA and CRC patient. Percentages of Treg cells are shown in CRA (n = 31), CRC (n = 31), and healthy controls (n = 24) (**B**), and in patients at early (CRA, n = 19; CRC n = 11) and advanced (CRA, n = 12; CRC n = 20) stages (**C**). Each datum point represents an individual sample. The horizontal line represents median values for each group.

Observations of Tregs in this study showed that percentage of Tregs was significantly increased in CRA and CRC patients (3.6%; 3.0% to 5.3%, *P =* 0.002; 4.4%; 2.6% to 5.9%, *P =* 0.002) as compared with healthy controls (2.8%; 2.2% to 3.6%). Although the percentage of Tregs was higher in CRC patients than that in CRA patients, no significant difference was observed between them (*P =* 0.36). In contrast to Th17 cells, the percentages of circulating Tregs were significantly higher in advanced stages than in early stages of both CRA and CRC patients (4.4%; 3.4% to 6.0% vs 3.5%; 2.5% to 4.7%, *P =* 0.047; 5.4%; 4.1% to 7.0% vs 2.5%; 1.2% to 3.8%, *P =* 0.003) (Figure 2).

### Seral concentrations of IL-1β, IL-6, IL-17A and IL-23 of CRA and CRC patients

**Figure 3:**
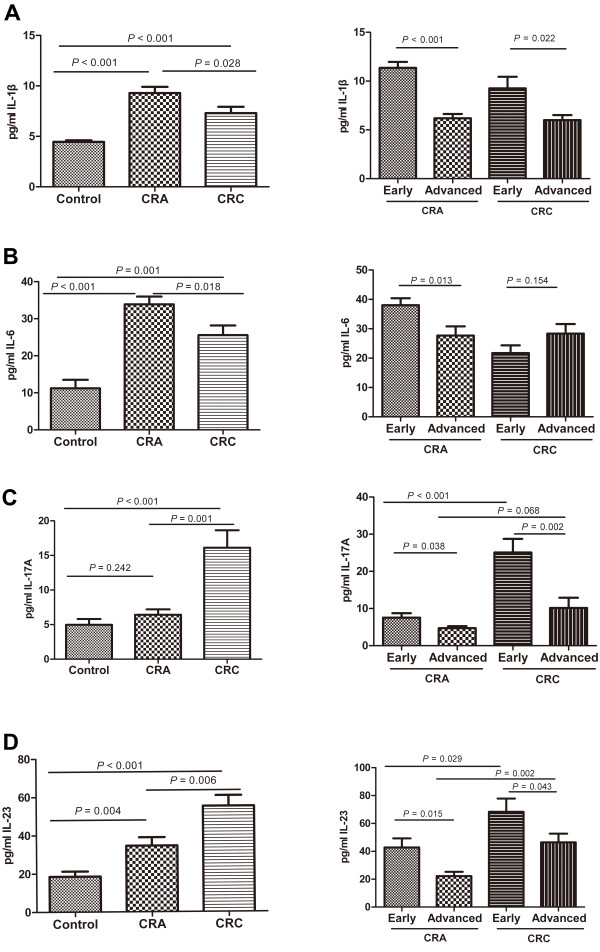
**Cytokine measurement in serum.** Concentrations of IL-1β (**A**),IL-6 (**B**),IL-17A (**C**) and IL-23 (**D**) in the serum of health donors (n = 16) and patients with CRA (n = 30; early stage, n = 18; advanced stage, n = 12) and CRC (n = 35; early stage, n = 14; advanced stage, n = 21) were measured by ELISA. The cytokine concentration (pg/ml) is expressed as mean ± SEM.

To investigate whether the changes of Th17 cells were accompanied by cytokine alterations, we then measured seral concentrations of IL-1β, IL-6, IL-17A and IL-23 in patients with CRA and CRC and healthy donors. As shown in Figure 3, concentrations of IL-17A and IL-23 were higher in CRA patients (IL-17A: 6.4 ± 0.8 pg/ml, *P =* 0.242; IL-23: 34.6 ± 4.4 pg/ml, *P =* 0.004 ) and highest in CRC patients (16.1 ± 2.5 pg/ml, *P <* 0.001; 55.2 ± 5.5 pg/ml, *P <* 0.001) as compared with healthy controls (5.0 ± 0.8 pg/ml; 18.6 ± 2.7 pg/ml). However, IL-1β and IL-6 were higher in CRC (IL-1β: 7.3 ± 0.6 pg/ml, *P <* 0.001; IL-6: 25.6 ± 2.6 pg/ml, *P =* 0.001) but highest in CRA (9.3 ± 0.6 pg/ml, *P <* 0.001; 33.9 ± 2.1 pg/ml, *P <* 0.001 ) as compared with healthy controls (4.6 ± 0.2 pg/ml; 11.2 ± 2.3 pg/ml). Following disease progression, we further found that IL-1β, IL-17A and IL-23 were significantly higher in early stages than in advanced stages of CRA patients (IL-1β: 11.4 ± 0.6 pg/ml vs 6.2 ± 0.4 pg/ml, *P <* 0.001; IL-17A : 7.6 ± 1.2 pg/ml vs 4.7 ± 0.5 pg/ml, *P =* 0.038; IL-23: 42.8 ± 6.5 pg/ml vs 22.2 ± 3.1 pg/ml, *P =* 0.015) and CRC patients (9.3 ± 1.2 pg/ml vs 6.0 ± 0.5 pg/ml, *P =* 0.022; 25.1 ± 3.7 pg/ml vs 10.2 ± 2.8 pg/ml, *P =* 0.002; 68.4 ± 9.5 pg/ml vs 46.5 ± 6.2 pg/ml, *P =* 0.043). Regardless of disease stages, IL-17A and IL-23 exhibited higher levels in CRC patients than in CRA patients (IL-17A: *P <* 0.001 or *P =* 0.068; IL-23: *P =* 0.029 or *P =* 0.002), whereas IL-6 concentration was increased in the advanced CRCs (28.3 ± 3.3 pg/ml vs 21.7 ± 2.6 pg/ml, *P =* 0.154) but decreased in the advanced CRAs (27.7 ± 3.1 pg/ml vs 38.0 ± 2.4 pg/ml, *P =* 0.013) as compared with early stage of each disease.

### Concentrations of IL-1β, IL-6, IL-17A, IL-21, IL-23 and TGF-β released from the cultured tissues

**Figure 4:**
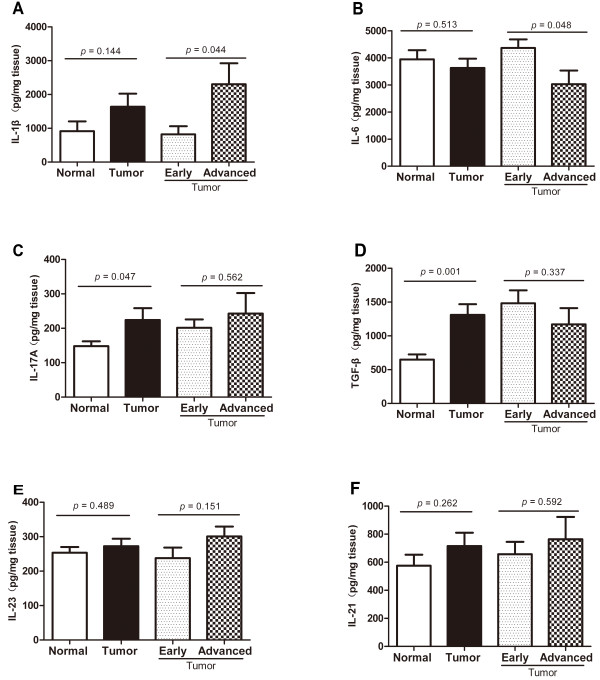
**Cytokine measurement in the supernatants of tissue culture.** Concentrations of IL-1β (**Α**),IL-6 (**B**),IL-17A (**C**), TGF-β (**D**), IL-23 (**E**),IL-21 (**F**) in the supernatant of colorectal normal tissues (n = 20) and CRC tissues(n = 20; early stage, n = 9; advanced stage, n = 11) of the same CRC patients were measured by ELISA. The cytokine concentration (pg/mg tissue) is expressed as mean ± SEM.

To reveal cytokine changes in tissues, we then measured concentrations of IL-1β, IL-6, IL-17A, IL-21, IL-23 and TGF-β in the supernatants of the cultured normal colorectal mucosa and CRC tissues. As shown in Figure 4, in comparison with normal mucosa, the tumor tissues released significantly higher amounts of IL-17A (224.2 ± 34.2 pg/mg tissue vs 148.3 ± 14.0 pg/mg tissue, *P =* 0.047) and TGF-β (1311.5 ± 157.6 pg/mg tissue vs 649.5 ± 75.6 pg/mg tissue, *P =* 0.001), but not IL-1β (*P =* 0.144), IL-21(*P =* 0.262), IL-23 (*P =* 0.489) and IL-6 (*P =* 0.513). However, we found that the tissues of the advanced CRC released significantly higher amount of IL-1β (2305.8 ± 618.8 pg/mg tissue vs 822.2 ± 240.9 pg/mg tissue, *P =* 0.044) than the tissues of the early CRC, while the release of IL-6 (4365.9 ± 317.1 pg/mg tissue vs 3026.2 ± 506.3 pg/mg tissue, *P =* 0.048) was significantly higher from the early CRCs than from the advanced CRCs. But IL-17A, IL-23, IL-21 and TGF-β released from the CRC tissues did not show a significant stage-associated change.

### Effect of IL-1β, IL-6 and TGF-β on the expansion of Th17 cells in PBMCs

**Figure 5:**
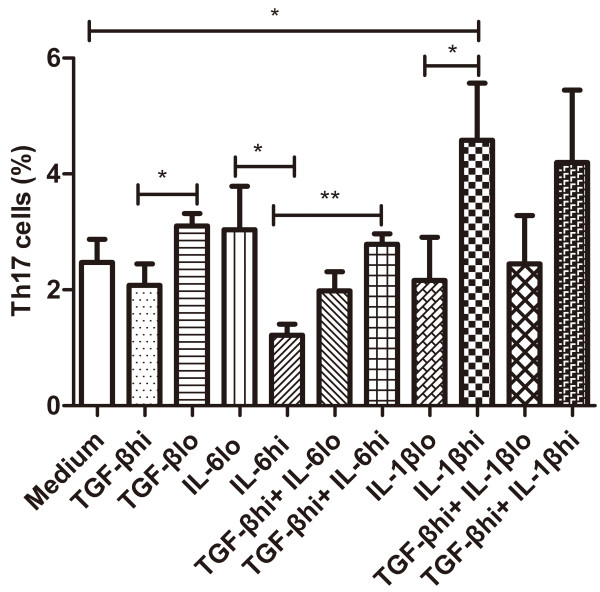
**Percentages of Th17 cell in the stimulated PBMCs in the presence of different cytokines.** Percentages of Th17 cells over the anti-CD3/CD28-stimulated PBMCs of the healthy donors following treatment with IL-1β^hi^ (25 ng/ml), IL-1β^lo^ (10 ng/ml), IL-6^hi^ (50 ng/ml), IL-6^lo^ (25 ng/ml), TGF-β^hi^ (5 ng/ml) and TGF-β^lo^ (0.5 ng/ml) are indicated. Results are expressed as mean ± SEM from six independent experiments. * P < 0.05, ** P < 0.01.

To further determine whether the changes of Th17 cells were the consequence of the alteration of IL-1β, IL-6 and TGF-β, we investigated the effects of IL-1β, IL-6 and TGF-β on the expansion of Th17 cells in PBMCs. The medium from PBMCs stimulated by anti-CD3 plus anti-CD28 with no cytokine treatment served as a control. Results in Figure 5 showed that 50 ng/ml IL-6 or 5 ng/ml TGF-β alone reduced the number of Th17 cells in comparison with 25 ng/ml IL-6 (*P =* 0.037) or 0.5 ng/ml TGF-β (0.5 ng/ml) (*P =* 0.038) alone. However, the combination of IL-6^hi^ (50 ng/ml) plus TGF-β^hi^ (5 ng/ml) significantly increased Th17 cell numbers as compared with IL-6 ^hi^ alone (*P <* 0.001). The high (hi) or low (lo) concentration of each cytokine was arbitrarily defined based on the significant effect of each cytokine on Th17 cell expansion. IL-1β^hi^ (25 ng/ml) increased the number of Th17 cells as compared with no cytokine control (*P =* 0.037) or IL-1β^lo^ (10 ng/ml) (*P =* 0.037), but IL-1β^hi^ plus TGF-β^hi^ slightly decreased the number of Th17 cells as compared with IL-1β^hi^ (*P =* 0.522). In addition, the changes of IL-17A level in the supernatants showed a similar trend with the changes of Th17 cell number, but did not show any statistical significance among different cytokine groups (data not shown).

## Discussion

The present study provided for the first time comprehensive data on the changes of Th17/Treg cells and cytokines IL-1β,IL-6,IL-17A,IL-21,IL-23 and TGF-β in the development and progression of CRC. It demonstrated in both CRA and CRC patients an increase of circulating Th17 cells in early stages and an increase of circulating Treg cells in advanced stages, and an increase of tumor infiltrating Th17 cells in advanced CRC tissues. The changes of Th17 cells along disease progression were accompanied by alterations of IL-1β, IL-6, IL-17A, IL-23 in serum and IL-1β, IL-6 in tumor tissues. *In vitro* studies further supported that the expansion of the Th17 cells were regulated by IL-1β, IL-6 and TGF-β in different combinations and/or concentrations.

Previously, the elevated percentage of Th17 cells were detected in blood, bone marrow, and spleen in mouse tumor models, and in peripheral blood, malignant ascites and tumor tissues in patients of advanced ovarian, pancreatic, renal cell carcinomas, melanoma, and breast and colon cancers [29,30]. In gastric cancer, there were elevations of Th17 cells in the peripheral blood as well as tumor-draining lymph nodes, both of which were associated with clinical stages of cancer development [31]. However, in ovarian cancer patients, the percentage of Th17 cells appeared higher in tumor but lower in peripheral blood or PBMCs and tumor-draining lymph nodes [32,33]. Our data showed that the frequency of Th17 cells was markedly increased in the circulation of both CRA and CRC patients, but became significantly lower as the diseases progressed to the advanced stages. Moreover, our observations were consistent with an animal study, in which the Th17 cells were increased in tumor tissues as disease progressed and reached to a maximal level in advanced tumors [29].

A previous study showed that IL-1β, IL-6 and TGF-β were involved in differentiation and expansion of the Th17 cells in ovarian cancers; IL-1β and IL-6 promoted, whereas TGF-β inhibited, Th17 cell expansion [32]. Our results showed that the changes in Th17 cell number along disease progression were accompanied by variations of IL-1β, IL-6 and IL-23 levels, suggesting the association of these cytokines with Th17 cell expansion. Given that the variation of cytokine levels, as seen in TGF-β, did not consistently follow the change of Th17 cells, we speculated the existence of an optimal level of these cytokines in regulating Th17 cell expansion. Indeed, we found that TGF-β^lo^, IL-6 ^lo^ or IL-1β^hi^ increased Th17 cell number in PBMCs. However,IL-6^hi^ plus TGF-β^hi^ unexpectedly promoted Th17 cell expansion, suggesting that a complex cytokine context, rather than the change of individual cytokines, is more likely involved in regulating Th17 cells. In the early stages, accumulation of Th17 cells in tumor tissues may be supported by high concentrations of TGF-β and IL-6. However, following tumor progression, high level of IL-1β, and possibly IL-23 as well, with the reduced levels of IL-6 and TGF-β may become supporting cytokine milieu for the expansion of Th17 cells in tumor tissues.

In this study, we detected the reduced level of IL-1β and increased frequency of Th17 cells in the circulation of CRCs as compared with that of CRAs. Since the IL-23 level is commonly elevated in the circulation of CRC patients, we speculated that IL-23 or other unknown factors, rather than IL-1β, may be more responsible for the expansion of Th17 cells in the circulation of CRC patients. Nevertheless, new studies are needed to test this hypothesis.

Previous reports showed that IL-21 promoted differentiation of human naïve CD4^+^T cells into Th17 cells [23]. IL-21 was found essential in patients of inflammatory bowel diseases (IBD) in promoting IL-17 production in anti-CD3/CD28-stimulated LPMCs, whereas IL-1β, IL-6, IL-23 and TGF-β exerted no effects [34]. However, our observation showed no significant difference in IL-21 level between normal tissues and tumor tissues or between early and advanced CRC tissues. This discrepancy may be interpreted by the difference in immunological mechanisms for regulating Th17 cells between IBD and cancer.

Previous studies showed that Th17 cells can be recruited into tumor microenvironment from the circulation [30]. These findings are supportive at least in part to our observations that, in advanced CRCs, the Th17 cells became reduced in the circulation but increased in tumor tissues. We cannot of course exclude another possibility that the Th17 cells were converted into Treg cells during tumor progression. The emerging evidence suggested that Th17 cells are of functional plasticity and can be converted into Treg cells *in vitro* and these cells cannot change back to Th17 cells even under the highly favorable conditions [35,36]. Nevertheless, new studies to test these two possibilities are warranted.

## Conclusions

Our study uncovers a unique change of Th17 cells and Treg cells and cytokines IL-1β, IL-6, IL-17A, and IL-23 along the progression of CRC. It further reveals that the Th17 cells are subjected to a complicated modulation by IL-1β,IL-6 and TGF-β. Clearly, the current study advances our understanding of the immunological mechanisms for CRC progression. It may thus help identify novel prognostic and therapeutic strategies for this disease.

## Abbreviations

CRA: Colorectal adenoma; CRC: Colorectal carcinoma; PBMCs: Peripheral blood mononuclear cells; NILs: Normal infiltrating lymphocytes; TILs: Tumor infiltrating lymphocytes; Treg: Regulatory T cell.

## Competing interests

The authors declare that they have no competing interests.

## Authors’ contributions

WJS and YBZ have made substantial contributions to the conception and design of the study, the acquisition, analysis and interpretation of data, and been responsible for critically drafting and revising the manuscript for all critical intellectual content. YBZ has supervised the entire study and given the final approval of the revision to be published. XKY, WJ, LCH, LYC, WXB, GH, FGS have made substantial contributions to sample collections, and data acquisition and interpretation. All authors read and approved the final manuscript.

## Pre-publication history

The pre-publication history for this paper can be accessed here:

http://www.biomedcentral.com/1471-2407/12/418/prepub
